# There is nothing new under the sun: ageism and intergenerational tension in the age of the COVID-19 outbreak

**DOI:** 10.1017/S1041610220000575

**Published:** 2020-04-14

**Authors:** Liat Ayalon

**Affiliations:** Louis and Gabi Weisfeld School of Social Work, Bar Ilan University, Ramat Gan, Israel

At the time of this writing, 203 countries and territories have been affected by the COVID-19 outbreak (Worldometer, 2020a). Older adults, in particular, are negatively impacted by this pandemic (Lipsitch *et al.*, 2020). Current estimates suggest that the COVID-19 mortality rate stands at 15% for those over the age of 80, but 0 for those under the age of 10 (Worldometer, 2020b). Hence, there is no doubt that age poses a major risk for COVID-19 mortality. At the same time, there are incidental reports of centenarians, who recovered from COVID-19, and of younger adults, who have not (Coffey and Oransky, 2020; Lanese and Writer, 2020). Moreover, the prevalence of younger people infected by the virus is higher than that of older adults (Surveillances, 2020). Hence, age alone is likely an insufficient criterion for predicting the direct medical impact of the outbreak.

In this commentary, I argue that the portrayal of all older adults as a homogenous, vulnerable group, rather than the use of a more refined discourse, which stresses the heterogeneity inherited in old age and the potential impact of the pandemic on society at large, has resulted in increased ageism and intergenerational tension, worldwide. Ageism is defined as stereotypes, prejudice, and discrimination toward people because of their age (Ayalon and Tesch-Römer, 2018; Officer and de la Fuente-Núñez, 2018). Although ageism can be both positive and negative (Ayalon and Tesch-Römer, 2018), in the context of the COVID-19 outbreak, we have seen an out surge of negative manifestations of ageism. Intergenerational tension, characterized as conflict between people of different generations, also has been intensified as a result of the outbreak and has fuelled the response to the pandemic. It is argued here that the psychosocial implications of the COVID-19 pandemic exacerbate and oftentimes supersede its direct medical impact, which to date, has received a substantial amount of research attention (World Health Organization, 2020).

From the get-go, the COVID-19 outbreak has been portrayed as “the problem of older adults” and a clear age division, separating young from old has been promoted (Zhou *et al.*, 2020). In China, where the COVID-19 outbreak had started, the tension between the generations has been manifested in anger toward older adults because of the refusal of some older adults to wear face masks (Eckersley, 2020). This is consistent with the prediction that at times of scarce resources, symbolic threats, manifested in disputes over values and beliefs, intensify (Stephan and Stephan, 2017). Overtime, the public focus has shifted to financial donations made by poor older adults, who sacrificed their living in an effort to support the fight against the pandemic. Both in the case of face masks and in the case of financial donations, older adults were portrayed as a separate, homogenous group in society, defined by its chronological age. In the former example, older adults were seen as a threat to society, as they “selfishly” refused to conform to current societal practices. In the latter example, on the other hand, older adults were presented as a selfless, yet vulnerable group, which is willing to risk its own being in order to help society at large.

In other countries, the division between young and old resulted in somewhat different manifestations, depending on the sociocultural background of the country and its ability to address the pandemic. In Israel, the Ministry of Defense had issued a statement that “the single most important insight… is to separate old people from young people. The single most lethal combination cocktail is when grandma meets her grandchild and hugs him” (Gross and TOI Staff, 2020). This statement explicitly argues for an age division between the generations and portrays intergenerational contact as THE problem. Following the same logic, in the UK, the first response to the outbreak was “business as usual.” In fact, the Prime Minister, Boris Johnson, had suggested that older adults over the age of 70 should self-isolate for a period of 4 months, while all other age groups continue as usual (Sparrow, 2020). A similar approach has been advocated in other countries, which had stressed the importance of socially isolating older adults, rather than the entire population (Armitage and Nellums, 2020). This approach was attributed to the fact that older adults already have their pensions and thus are not likely to be impacted financially by social isolation. Moreover, older adults have “already lived their lives,” and now it is time for them to step down. Whereas protecting those most vulnerable to the virus is commendable, compromising older adults’ autonomy and disregarding their social contribution and social needs are ageist.

These messages are ageist, as they take chronological age as a sole criterion, and automatically equate older age with vulnerability, dependency, and limited contribution. This ignores the diversity that is present especially in old age and regards all people over a certain age as a homogenous group (Lowsky *et al.*, 2014). We know from past research that telling older adults that they are vulnerable, unable or unfit impairs their performance and impacts their health and wellbeing (Hausdorff *et al.*, 1999). Furthermore, the way older adults think about their own aging has a direct association with their morbidity and mortality prospects, with those who think of their aging in a negative way, being more likely to suffer from a variety of health and mental health conditions and even die before those who think more positively about their aging (Ayalon, 2016; Levy and Myers, 2005; Pietrzak *et al.*, 2016; Robertson *et al.*, 2016).

The division between young and old and the portrayal of older adults as the main at-risk group have resulted in younger people feeling invincible, thinking “this is not their illness.” In the USA, Germany, and other countries, younger people have celebrated the COVID-19 outbreak in “Corona parties,” thinking they are immune (Casey, 2020). The hashtag #Boomerremover has become popular in the USA and other countries, stressing the growing division and animosity between the generations (Godfrey, 2020). Hence, the reliance on chronological age as a marker of risk has resulted in adverse societal consequences.

The transition from seeing older and younger people as two separate groups to sacrificing older adults for the greater good of society has been quick. In Israel, the former CEO of the Ministry of Health had stated that “for very few people, whose expected lifespan is not very high, you do not ruin a country.” He further said that the state should “sacrifice these people” (Magen, 2020). Similar messages have been expressed in other parts of the world. For instance, in the USA, Texas Lieutenant Governor, Dan Patrick, had said that he would rather die than damage the US economy, adding that “lots of grandparents would agree with him” (Becket, 2020). These statements are in line with past research that has found that older adults are expected to succumb and give their place to the younger generations. They are also expected not to consume too much, simply because of their chronological age (North and Fiske, 2013).

Realistic threats, which represent disputes over material assets, occur when resources become scarce (Stephan and Stephan, 2017). With the growing impact of the pandemic, the rationing of treatment has become a continuous debate (Swiss Academy of Medical Sciences, 2020; Truog *et al.*, 2020). With some countries, explicitly using age as a criterion for the allocation of treatment. For instance, the Italian College of Anesthesia had issued a statement that it might be necessary to put an age limit on accessing intensive care in order to save resources for those who among other things, have more years of life left (Vergano *et al.*, 2020). Consistently, in Spain, which has been badly hit by the virus, some older adults were completely abandoned to die alone in their beds, following the flee of their care staff. This is a direct outcome of the rationing of treatment by chronological age and the perception of older adults as being of a lesser value and a burden on the economy and the health care system.

During the COVID-19 outbreak, intergenerational solidarity has been brutally negated and social distancing (Mahase, 2020), rather than physical distancing has become the norm. The pandemic, however, has occurred in the context of already highly divided societies, around multiple issues presented as generational in nature. The Brexit in the UK (Banaji and Mejias, 2018), voting rights for those whose life expectancy is lower than 18 years in Germany (Maschiach, 2019), or the climate change have been depicted as sources of intergenerational divide with the younger generations blaming older adults for “stealing their future” (Morrison, 2020). The social isolation and abandonment of older adults are not new and have been noted in other emergency situations, such as the Chicago 1995 heat wave, for instance (Klinenberg, 2015). In fact, the higher vulnerability of older adults in the face of emergency situations has been attributed to their limited social capital, defined by the quantity and quality of informal (e.g. friends and family) and formal (e.g. community and volunteers) social ties (Durant Jr, 2011). When social isolation and intergenerational distancing are promoted as the norm, as in the case in the current pandemic, older adults’ ability to face natural atrocities deteriorates even further.

## What can we do better?

Chronological age should not be used for the allocation of goods and services and should not be a sole criterion for determining people’s vulnerabilities, prognosis, or treatment options. There is a high variability in old age, which in fact becomes even greater as people grow older (Lowsky *et al.*, 2014). This should be acknowledged and guide the response to the COVID-19 outbreak. Decisions concerning the rationing of treatment should not be based solely on chronological age. Instead, people’s values and preferences as well as short-term outcomes should be taken into consideration (Swiss Academy of Medical Sciences, 2020). Moreover, old age should not be automatically equated with vulnerability and dependency, as this results in substantial harm to people’s perceptions of their own aging selves and intensifies the division between the generations. An alternative framing, which portrays the negative implications of the COVID-19 outbreak on all member of society, regardless of their chronological age, should be advocated.

The divisive language of “us” vs. “them” or young vs. old splits societies and has a malicious impact not only on older adults, but also on younger adults and on our already fragile social fabric (Ayalon and Tesch-Römer, 2018; Scharf et al., 2020). We should also avoid using terms such as social distancing and instead use the more neutral term physical distancing. The latter term implies that while for our safety, physical contact might be minimal to non-existent, social contact should be maintained and even strengthened. In fact, social capital (Durant Jr, 2011), including intergenerational contact, is a major asset in emergency situations and its absence makes older adults particularly vulnerable.

Finally, the most vulnerable members of our society, including older adults in long-term care facilities and those with severe physical and cognitive impairments, face substantial threats to their autonomy, unrelated to the current outbreak. It is our duty to ensure that the autonomy of the most vulnerable members of society is not hampered and that their voices are heard. Older people’s rights should not be compromised, and human rights should not be differentially allocated based on chronological age (United Nations Human rights – Office of the High Commissioner, 2020; Age Platform Europe, 2020).
